# The use of a proposed updated EARL harmonization of ^18^F-FDG PET-CT in patients with lymphoma yields significant differences in Deauville score compared with current EARL recommendations

**DOI:** 10.1186/s13550-019-0536-3

**Published:** 2019-07-25

**Authors:** John Ly, David Minarik, Lars Edenbrandt, Per Wollmer, Elin Trägårdh

**Affiliations:** 1 Department of Radiology, Kristianstad Hospital, Kristianstad, Sweden; 2 Department of Translational Medicine, Lund University, Malmö, Sweden; 3 Radiation Physics, Skåne University Hospital and Lund University, Malmö, Sweden; 4 Department of Clinical Physiology and Nuclear Medicine, Sahlgrenska University Hospital, Region Västra Götaland, Gothenburg, Sweden; 5 Department of Clinical Physiology and Nuclear Medicine, Skåne University Hospital, Malmö, Sweden; 6 Wallenberg Centre for Molecular Medicine, Lund University, Lund, Sweden

**Keywords:** Deauville score, Reconstruction algorithm, Lymphoma, EARL, PET

## Abstract

**Background:**

The Deauville score (DS) is a clinical tool, based on the comparison between lesion and reference organ uptake of ^18^F-fluorodeoxyglucose (FDG), used to stratify patients with lymphoma into categories reflecting their disease status. With a plethora of positron emission tomography with computed tomography (PET-CT) hard- and software algorithms, standard uptake value (SUV) in lesions and reference organs may differ which affects DS classification and therefore medical treatment. The EANM Research Ltd. (EARL) harmonization program from the European Association of Nuclear Medicine (EANM) partly mitigates this issue, but local preferences are common in clinical practice. We have investigated the discordance in DS calculated from patients with lymphoma referred for ^18^F-FDG PET-CT reconstructed with three different algorithms: the newly introduced block-sequential regularization expectation-maximization algorithm commercially sold as Q. Clear (QC, GE Healthcare, Milwaukee, WI, USA), compliant with the newly proposed updated EARL recommendations, and two settings compliant with the current EARL recommendations (EARL_lower_ and EARL_upper_, representing the lower and upper limit of the EARL recommendations).

**Methods:**

Fifty-two patients with non-Hodgkin and Hodgkin lymphoma were included (18 females and 34 males). Segmentation of mediastinal blood pool and liver were semi-automatically performed, whereas segmentation of lesions was done manually. From these segmentations, SUV_max_ and SUV_peak_ were obtained and DS calculated.

**Results:**

There was a significant difference in DS between the QC algorithm and EARL_lower_/EARL_upper_ (*p* < 0.0001 for both) but not between EARL_lower_ and EARL_upper_ (*p* = 0.102) when SUV_max_ was used. For SUV_peak_, there was a significant difference between QC and EARL_lower_ (*p* = 0.001), but not for QC vs EARL_upper_ (*p* = 0.071) or EARL_lower_ vs EARL_upper_ (*p* = 0.102). Five non-responders (DS 4–5) for QC were classified as responders (DS 1–3) when EARL_lower_/EARL_upper_ was used, both when SUV_max_ and SUV_peak_ were investigated.

**Conclusion:**

Using the proposed updated EARL recommendations compared with the current recommendations will significantly change DS classification. In select cases, the discordance would affect the choice of medical treatment. Specifically, the current EARL recommendations were more often prone to classify patients as responders.

## Background

Over the years, there have been multiple advances in positron emission tomography with computed tomography (PET-CT) regarding both hard- and software. The new developments, such as introduction of time-of-flight, point-spread-function, smaller voxels, respiratory gating, silicon (Si) photomultiplier (PM) detectors and block-sequential regularized expectation maximization (BSREM) reconstruction algorithms (commercially sold as Q. Clear (QC), GE Healthcare, Milwaukee, WI, USA), have all contributed to a better image quality, improved small lesion detectability and more accurate quantification of radiopharmaceutical uptake [1].

In patients with Hodgkin and non-Hodgkin lymphoma, ^18^F-fluorodeoxyglucose (FDG) PET-CT has become the standard procedure in the staging, monitoring and restaging of disease. During therapy assessment at mid-treatment and after completion of chemotherapy, the Deauville score (DS) is recommended to discriminate between responders and non-responders [2, 3]. DS is a 5-point scale where the lesion with the most intense uptake is compared to the physiological uptake in the mediastinal blood pool and the liver. Responders are usually defined as DS 1–3 and non-responders as DS 4–5 [4–6].

The new developments in PET have been shown to affect the maximum standardized uptake value (SUV) in lesions [7]. It can therefore be suspected that a patient being examined on different PET-CT scanners with different hard- and software as well as with different acquisition parameters might receive different DS. To overcome this issue, the EANM Research Ltd. (EARL) harmonization programme from the European Association of Nuclear Medicine (EANM) has set up recommendations on how to perform PET imaging for oncologic purpose, including harmonization of the patient preparation, scan acquisition, image processing and interpretation of images, in order to be able to compare results from different PET-CT scanners [8]. This harmonization, however, does not take the most modern applications of PET hard- and software in consideration and might underestimate DS in small lesions. Recently, there has been a proposal for updating the EANM/EARL recommendations to include modern PET-CT equipment [9].

The aim of this study was to investigate whether using a novel state-of-the-art SiPM-based PET-CT with QC reconstruction (that complies with the newly proposed updating of the EANM/EARL recommendation) may affect DS compared with reconstructions meeting the current EANM/EARL harmonizing standard in patients with lymphoma, regarding both SUV_max_ and SUV_peak_.

## Methods

### Patients

In this retrospective study, 57 patients who underwent clinical ^18^F-FDG PET-CT between November 2017 and March 2018 or August 2018 to October 2018 at Skåne University Hospital in Lund or Malmö, Sweden, were included initially. Patients admitted for baseline PET, mid-treatment (interim) PET (i-PET), end-of-treatment PET (EoT-PET) and suspicion of recurrence were included. Four patients did not have any discernible lesion on CT and one patient had a history of lymphoma but not at the time of the examination, which was performed for other reasons. These five patients were excluded, leaving 52 patients in the study.

### PET-CT acquisition and reconstruction parameters

Three Discovery MI (GE Healthcare, Milwaukee, WI, USA) PET-CT systems were used for image acquisition. The systems were configured with four rings of detector blocks with lutetium yttrium oxyorthosilicate crystals coupled to an array of SiPM. The PET-detector has a transaxial field of view of 70 cm, an axial field of view of 20 cm and an overlap of 24% between bed positions. The sensitivity, according to NEMA standards, was 13 cps/kBq. The PET system was combined with a 128 slice CT.

All patients received an intravenous injection of 4 MBq/kg body weight of ^18^F-FDG with an accumulation time of 60 min before imaging and after at least 4 h of fasting and a glucose level ≤ 10 mM. If no contraindications existed, the patients were administered with beta-blockers before the examination. Patients were scanned from the inguinal region to the base of the skull. Acquisition time was 1.5 min per bed position. CT images were acquired for attenuation correction and anatomic correlation of the PET images. A diagnostic CT with intravenous and oral contrast or a low-dose CT without contrast was performed. In our clinical routine, a low-dose is performed if a previous diagnostic CT has been performed within 4 weeks. For diagnostic CTs, tube current modulation was applied by adjusting the tube current for each individual with a noise index of 42.25 and a tube voltage of 100 kV. For low-dose CT, the tube voltage was 120 kV with a noise index of 45. If a diagnostic CT was performed, it was used for attenuation correction (delayed venous phase of intravenous contrast). The same CT was used for attenuation correction for all PET reconstructions. The adaptive statistical iterative reconstruction technique (ASiR-V) was applied for all CT reconstructions.

In order to compare the reconstruction algorithms, we reconstructed different data series, where the selected reconstruction parameters were based on phantom measurements in accordance with the EARL standard [10]. The EARL standard defines lower and upper limits for the resolution recovery coefficient (RRC) for different sized spheres in the NEMA-phantom and limits of the noise level. Two reconstructions were made corresponding to the lower (EARL_lower_) and upper level (EARL_upper_) of the RRCs. The ordered subset expectation maximization (OSEM) algorithm was used without resolution recovery or time of flight. For the upper level, the images were reconstructed with 4 iterations, 16 subsets and a Gaussian post filter with a FWHM of 5 mm. For the lower-level images, the reconstructions were performed with 3 iterations, 8 subsets and a post-filter with 7 mm FWHM. A new EARL standard has been proposed where the RRC limits have been substantially increased to accommodate modern systems [9]. One reconstruction was made which yields EARL results that fall near the upper level of the new EARL standard. The QC reconstruction algorithm was used, with a beta value of 500 [11]. The slice thickness for all three reconstructions was 2.79 mm, the matrix and pixel size were 192 × 192 and 3.64 mm for the EARL reconstructions and 256 × 256 and 2.73 mm for the QC reconstruction.

### Image analysis

A machine learning method described previously [12] was used to segment the liver and the mediastinal blood pool (thoracic part of the aorta) in the CT images. One radiology resident and one specialist in radiology and nuclear medicine corrected the automated segmentations when needed. Focal lesions within the liver were not included in the segmentation. The segmentations were then eroded by 3 voxels in all directions in order to avoid the edges of the respective organs. The EARL reconstructions were regridded to the same pixel size as the QC reconstruction prior to the erosion operation, giving an erode kernel of 8.2 × 8.2 × 8.4 mm for all reconstructions.

Lymphoma lesions were manually segmented in the CT images by the two physicians described above. The PET image could be overlaid to help segmentation in case of registration mismatch between the CT and PET, wherein such cases segmentations were slightly outside the CT lesion in order to include the lesion SUV_max_.

The SUV_max_ and SUV_peak_ of the liver, blood pool and lesions were calculated using the segmentations made in the CT image translated to the corresponding locations in the PET images. Lesion SUV_max_ and SUV_peak_ were then compared to SUV_max_ and SUV_peak_ in the liver and blood pool in order to assign a DS.

### Statistical analysis

Continuous patient parameters are presented as mean ± standard deviation (SD) and range and categorical variables as a percentage (%). A Friedman test comparing the DS obtained from the three different reconstruction algorithms was performed for SUV_max_ and SUV_peak_, respectively. Significant *p* value was set at *p* < 0.05. Post hoc analysis with Wilcoxon signed-rank test was conducted with a Bonferroni correction applied, resulting in a significance level set at *p* < 0.0167. All statistical tests were performed using IBM SPSS version 25 (IBM, Armonk, NY, USA).

## Results

### Patients

Fifty-two patients with lymphoma were enrolled (16 Hodgkin’s lymphoma, 1 Burkitt’s lymphoma, 2 B cell lymphoma (subtype unavailable), 23 diffuse large B cell lymphoma, 6 follicular lymphoma, 2 mantle cell lymphoma, 1 peripheral T cell lymphoma and 1 anaplastic large cell lymphoma). There were in total 10 baseline PET, 13 i-PET, 26 EoT-PET examinations and 5 suspicion of recurrence. Two patients underwent both i-PET and EoT-PET, resulting in *n* = 54 PET-CT examinations. Patients were aged between 17 and 83 years of which 35% were women. The mean (± SD) weight was 79 ± 16 kg (range 46–137 kg) and the mean BMI was 25.8 ± 4.6 (range 16.9–39.6). The mean administrated ^18^F-FDG was 4.0 ± 0.15 MBq/kg (range 3.0–4.3 MBq/kg) and the mean accumulation time was 62 ± 4 min (range 54–78 min).

### Quantitative analysis

None of the patients were classified as DS 1. Table 1 shows the number of patients classified as DS 2–5 for the three different reconstruction methods using SUV_max_ and SUV_peak_.

**Table 1:** Classifications of DS for the three different reconstruction methods using SUV_max_ (A–C) and SUV_peak_ (D–F)

A		EARL_lower_ (SUV_max_)				
		DS2	DS3	DS4	DS5	Total
QC (SUV_max_)	DS2	14	0	0	0	14
	DS3	8	5	0	0	13
	DS4	1	2	3	0	6
	DS5	0	1	6	14	21
Total		23	8	9	14	54
B		EARL_upper_ (SUV_max_)				
		DS2	DS3	DS4	DS5	Total
QC (SUV_max_)	DS2	13	1	0	0	14
	DS3	6	7	0	0	13
	DS4	1	3	2	0	6
	DS5	0	0	6	15	21
Total		20	11	8	15	54
C		EARL_upper_ (SUV_max_)				
		DS2	DS3	DS4	DS5	Total
EARL_lower_ (SUV_max_)	DS2	20	3	0	0	23
	DS3	0	7	1	0	8
	DS4	0	1	7	1	9
	DS5	0	0	0	14	14
Total		20	11	8	15	54
D		EARL_lower_ (SUV_peak_)				
		DS2	DS3	DS4	DS5	Total
QC (SUV_peak_)	DS2	20	0	0	0	20
	DS3	3	4	0	0	7
	DS4	0	3	6	0	9
	DS5	0	0	5	13	18
Total		23	7	11	13	54
E		EARL_upper_ (SUV_peak_)				
		DS2	DS3	DS4	DS5	Total
QC (SUV_peak_)	DS2	18	2	0	0	20
	DS3	2	4	1	0	7
	DS4	0	4	4	1	9
	DS5	0	0	5	13	18
Total		20	10	10	14	54
F		EARL_upper_ (SUV_peak_)				
		DS2	DS3	DS4	DS5	Total
EARL_lower_ (SUV_peak_)	DS2	20	3	0	0	23
	DS3	0	6	1	0	7
	DS4	0	1	9	1	11
	DS5	0	0	0	13	13
Total		20	10	10	14	54

For SUV_max_ calculations, the Friedman test resulted in *p* < 0.0001. Five (9.3%) QC non-responders (DS 4–5) became responders (DS 2–3) with EARL_lower_ and/or EARL_upper_. When comparing EARL_lower_ with EARL_upper_, one patient changed from DS 3 to DS 4 and one patient changed from DS 4 to DS 3. No other patient changed from non-responder to responder or vice versa. Discordance in DS occurred in 18 cases (33.3%) when comparing QC with EARL_lower_, in 17 cases (31.5%) when comparing QC with EARL_upper_, and in 6 cases (11.1%) when comparing EARL_lower_ with EARL_upper_. Discordant lesions were consistently downscaled in DS with either EARL_lower_ or EARL_upper_ compared to QC, except for one patient that was upscaled from DS 2 (QC) to DS3 (EARL_upper_). Wilcoxon tests resulted in QC vs EARL_lower_ *p* < 0.0001, QC vs EARL_upper_ *p* < 0.0001 and EARL_lower_ vs EARL_upper_ *p* = 0.102 (not significant).

For SUV_peak_ calculations, the Friedman test resulted in *p* = 0.003. Five (9.3%) QC non-responders became responders with EARL_lower_ and/or EARL_upper_. When comparing EARL_lower_ with EARL_upper_, one patient changed from DS 3 to DS 4 and one patient changed from DS 4 to DS 3. No other patient changed from non-responder to responder or vice versa. Discordance in DS occurred in 11 cases (20.4%) when comparing QC with EARL_lower_, in 15 cases (27.8%) when comparing QC with EARL_upper_ and in 6 cases (11.1%) when comparing EARL_lower_ with EARL_upper_. Discordant lesions were consistently downscaled in DS with either EARL_lower_ or EARL_upper_ compared to QC, except for four patients. Two patients were upscaled from DS 2 (QC) to DS3 (EARL_upper_), one patient from DS 3 (QC) to DS 4 (EARL_upper_) and one patient from DS 4 (QC) to DS 5 (EARL_upper_). Wilcoxon tests resulted in QC vs EARL_lower_ *p* = 0.001, QC vs EARL_upper_ *p* = 0.071 (not significant) and EARL_lower_ vs EARL_upper_ *p* = 0.102 (not significant).

**Fig. 1:**
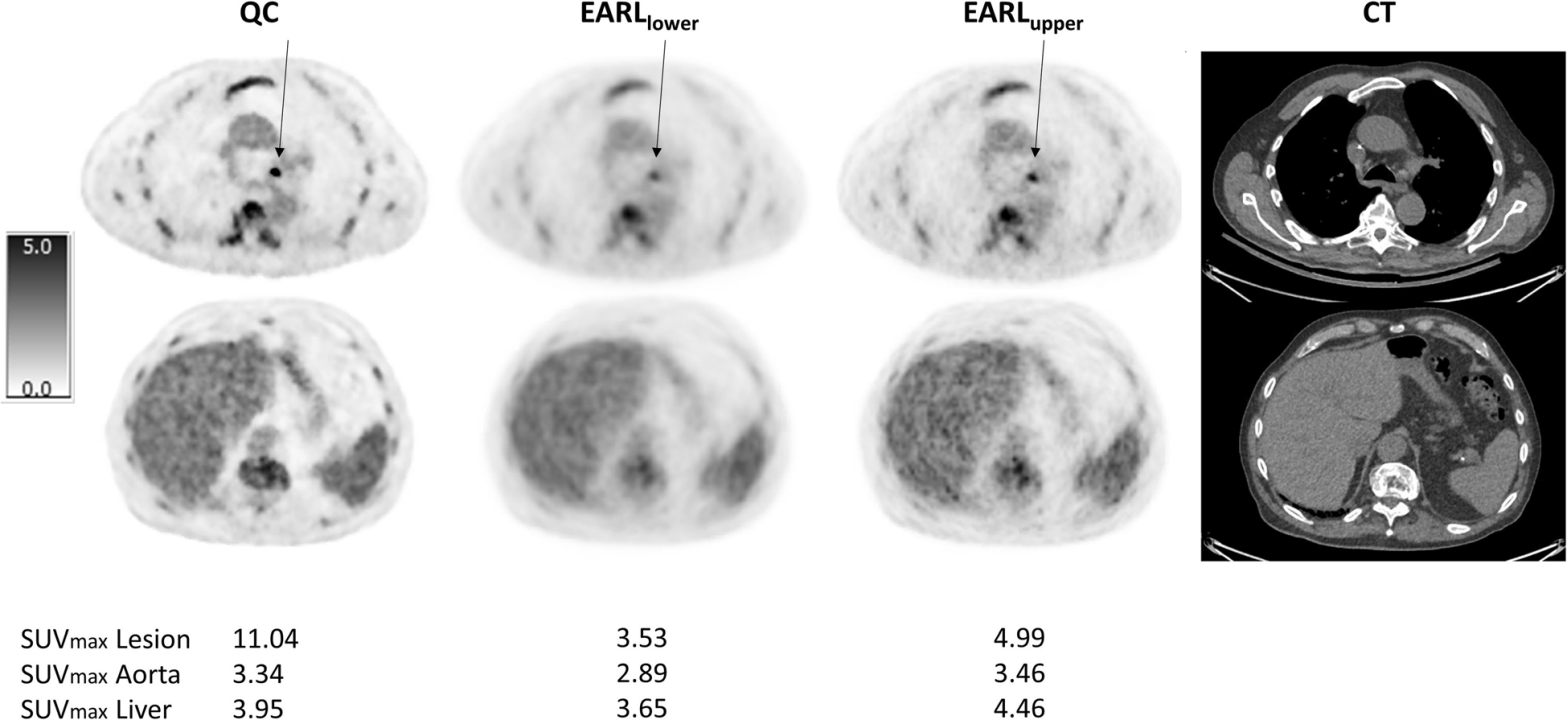
Major discordance in DS. Representative example of patient who had major discordance between QC, EARL_lower_ and EARL_upper_ DSs. This patient was classified as DS 5 on QC images, DS 3 on EARL_lower_ images and DS 4 on EARL_upper_ images. Transversal images in the thorax (aorta and lesion (indicated with an arrow) with highest SUV_max_)—upper row—and in the abdomen (liver)—lower row—for the three different reconstructions including the corresponding CT are shown

**Fig. 2:**
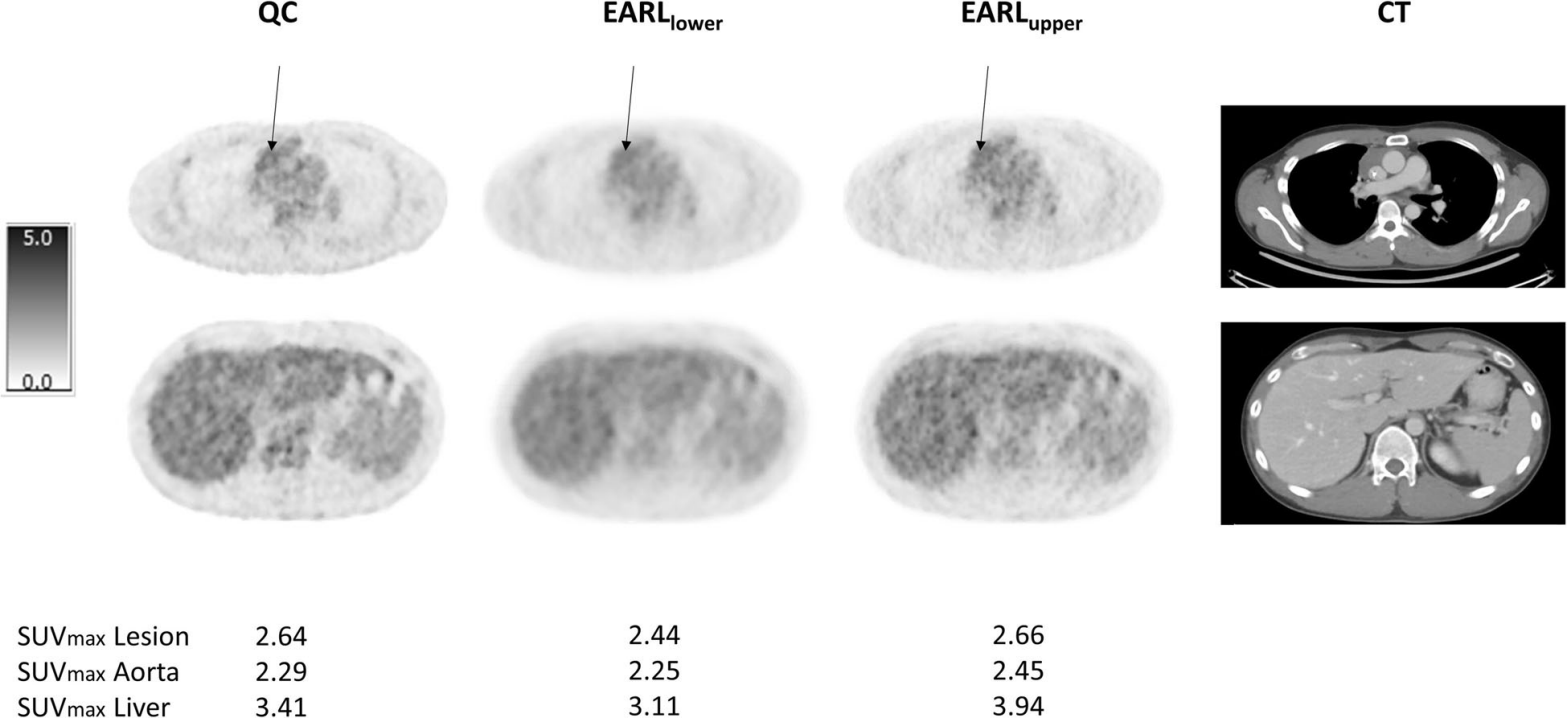
Good concordance in DS. Representative example of patient who had good concordance between QC, EARL_lower_ and EARL_upper_ DSs. This patient was classified as DS 3 on all reconstruction settings. Transversal images in the thorax (aorta and lesion (indicated with an arrow) with highest SUV_max_)—upper row—and in the abdomen (liver)—lower row—for the three different reconstructions including the corresponding CT are shown

**Fig. 3:**
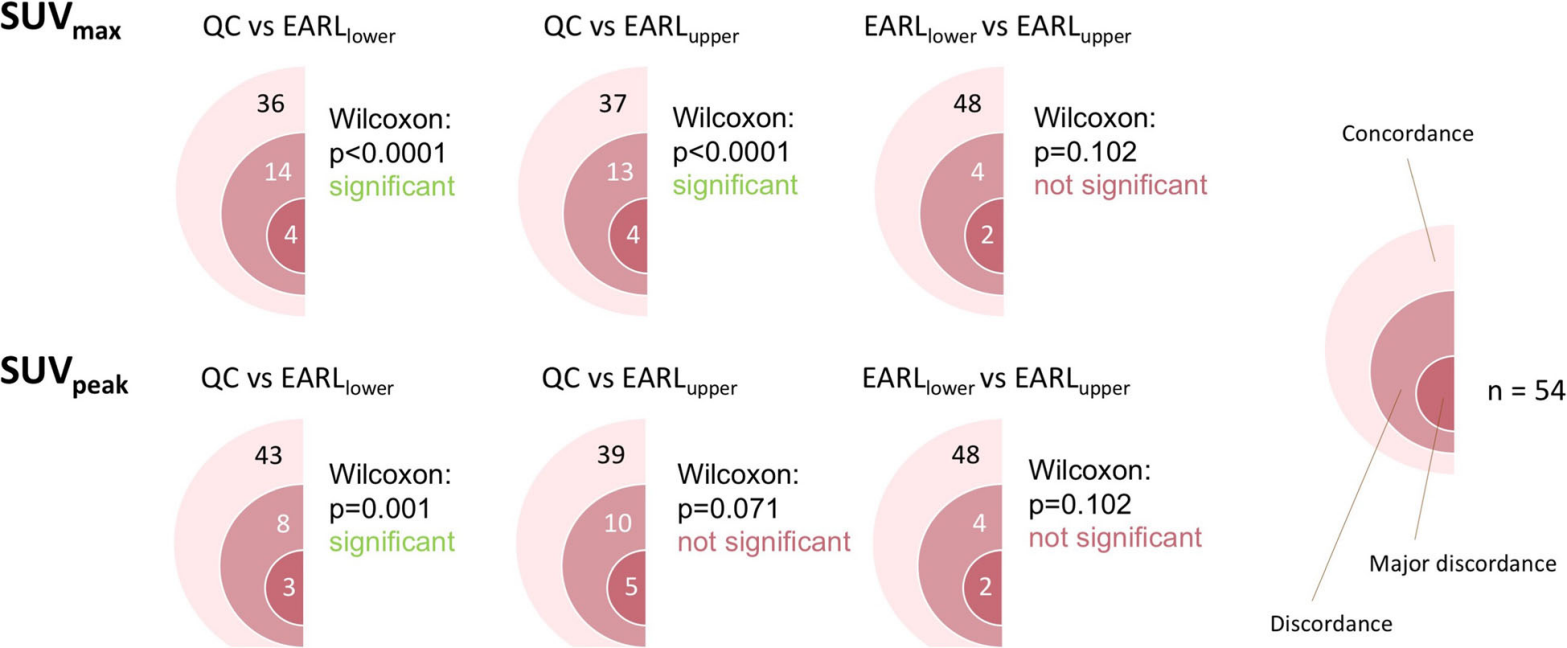
Diagrams of pairwise comparisons between reconstruction algorithms. Numbers in coloured semi-circles represent *n* cases that had concordance, discordance and major discordance respectively when comparing reconstruction algorithms pairwise. For SUV_max_, post hoc analysis with Wilcoxon signed-rank test with Bonferroni correction, QC compared to E_lower_ and E_upper_ respectively yielded significant *p* value but not for E_lower_ compared to E_upper_. For SUV_peak_, only QC compared to E_lower_ yielded significant *p* value

Figure 1 shows an example of a patient with major discordance between QC, EARL_lower_ and EARL_upper_ DS. Figure 2 shows an example of a patient with good concordance for DS between the different reconstruction settings. Figure 3 shows the ratios between concordance, discordance and major discordances when comparing the reconstruction algorithms pairwise.

## Discussion

The latest proposed update to the EARL recommendations accommodates modern PET technologies, in particular time-of-flight and point spread function (PSF) [6]. Our study compares datasets that are compatible with both current and the proposed update to the EARL recommendations. The introduction of time-of-flight and PSF have shown to have minimal effect on the liver and mediastinal uptake [10], but new hardware and reconstruction algorithms provide higher SUV in small lesions [4], and therefore affect DS classification. Our findings show that if the proposed update to the EARL recommendations is accepted, it will have an impact on DS and therapy response evaluation. The studies behind the recommendations on DS and treatment evaluation are performed on older generations of PET-CT scanners, and it is not known whether the DS obtained from new state-of-the-art PET-CT scanners will have an impact on patient outcome in large cohorts.

Investigation of whether the choice of reconstruction method affects DS has recently been performed by Enilorac et al. [13], comparing one dataset with unfiltered PSF (Siemens HD) and one where a 6-mm Gaussian filter was applied to PSF images to match the EARL requirements. The proportion of major discordances was comparable to our findings for SUV_max_ but our conclusions differ. In their study of 126 patients, no difference in progression-free survival and overall survival was seen depending on the reconstruction method, when patients were classified as responders or non-responders. However, they analysed i-PET and EoT-PET separately, yielding small groups with only a few patients classified differently depending on the reconstruction method.

There are different aspects on DS that should be considered, such as how SUV in reference organs are measured, the cut-off for DS 5 and how to handle patients with a higher SUV in the mediastinum compared with liver. In our study, we used automatic segmentation of liver and mediastinum [6]. The edges around the liver and aortic wall were automatically truncated to avoid uptake from adjacent structures and the vessel wall. Segmentations were manually corrected when needed. This method increases the likelihood of obtaining the true SUV_max_. When manual ROIs are placed in reference organs, there is an apparent risk of missing the true SUV_max_. In a couple of the patients, we found the mediastinal SUV_max_ to be higher than the liver SUV_max_. This was confirmed with manual ROI measurements (data not shown). There is no support in the literature or guidelines on how these cases should be managed in terms of DS classification. However, in our study, no patient had lesion uptake that was between uptake in the mediastinum and in the liver. There is no consensus where the cut-off point should be for DS 5, and both a limit of two or three times the maximum uptake in the liver has been proposed [4]. We classified DS 5 as two times the maximum uptake in the liver. There were few major discordances in DS (i.e. when a non-responder (QC) is reclassified as responder (EARL_lower_/EARL_upper_)) between reconstruction methods, which has clinical significance in terms of treatment strategy. If a worst-case scenario is preferred, then using settings that adhere to the newly proposed EARL recommendation is more suitable.

We included baseline exams in order to increase the study population, although DS is normally not calculated in baseline examinations. In theory, a follow-up scan could look like a baseline scan. In the retrospective analysis, baseline exams did not show any major discordances: for SUV_max_, there were 2 discordances, and for SUV_peak_, there were also 2 discordances. If all baseline scans were removed from the study, the results would show an even higher percentage of discordances across all pairwise comparisons between reconstruction algorithms.

No solid recommendation of how to obtain DS exists, although SUV_max_ appears to be the most commonly used method. In this study, we investigated the use of both SUV_max_ and SUV_peak_, as proposed both by Barrington et al. [4] and the newly proposed EARL recommendations [9]. SUV_max_ is more noise dependent [14]; therefore, SUV_peak_ is a more stable measure. This was also true in our study, where we did not find any significant differences in DS between QC and EARL_upper_. However, SUV_peak_ requires a lesion of more than 1 cm in order to be relevant. SUV_max_, on the other hand, has been shown to be unreliable in sub-centimetre lesions when PSF is used [15]. There is no standard definition of SUV_peak_ calculation which may be seen in differing implementations of SUV_peak_ calculations in various software. A harmonization across vendors is desirable to further increase its reproducibility.

### Limitations

In this study, we included all patients with lymphoma, regardless of the indication for the PET-CT examination. In clinical routine, DS is only used for therapy assessment and not for initial staging/baseline. However, in order to increase the number of patients and the range of included DS, also, patients referred for baseline PET-CT were included. Despite this, we recognize the limitation of the study due to its small sample size and its monocentric nature.

Although we have showed considerable differences in DS between the reconstruction algorithms, it remains to be proven which reconstruction algorithm has the most favourable outcome for the patients. The type of lymphoma and the intensity of stage-adapted chemotherapy adds further complexity to the outcome.

1It would be of interest to compare the upper and lower limits of the newly proposed EARL recommendations, but for our PET-CT system, longer acquisition times are necessary to reach the new upper limit, which was not feasible for the current study.

## Conclusions

There is a significant difference in DS classification when comparing the proposed update to EARL recommendations and the current recommendations. In select cases, the discordance would affect the choice of medical treatment. Specifically, the current EARL recommendations were more often prone to classify patients as responders compared with the recently proposed EARL update. Further studies are needed in order to prove which reconstruction algorithm is suitable for assessing patient outcome.

## Data Availability

The datasets used and/or analyzed during the current study are available from the corresponding author on reasonable request.

## Abbreviations

ASiR-V: Adaptive statistical iterative reconstruction technique
BSREM: Block-sequential regularized expectation maximization
DS: Deauville score
EANM: European Association of Nuclear Medicine
EARL: EANM Research Ltd.
EoT-PET: End-of-treatment PET
FDG: Fluorodeoxyglucose
i-PET: Interim-PET
OSEM: Ordered subset expectation maximization
PET-CT: Positron emission tomography/computed tomography
PM: Photomultiplier
PSF: Point-spread-function
QC: Q. Clear
RRC: Resolution recovery coefficient
SD: Standard deviation
Si: Silicon
SUV: Standardized uptake value
